# Extracellular cathepsin L stimulates axonal growth in neurons

**DOI:** 10.1186/s13104-017-2940-y

**Published:** 2017-11-23

**Authors:** Chihiro Tohda, Michihisa Tohda

**Affiliations:** 1 Division of Neuromedical Science, Institute of Natural Medicine, University of Toyama, 2630 Sugitani, Toyama, 930-0194 Japan; 2 Division of Medicinal Pharmacology, Institute of Natural Medicine, University of Toyama, 2630 Sugitani, Toyama, 930-0194 Japan

**Keywords:** Axonal growth, Cortical neurons, Spinal cord neurons, Cathepsin L

## Abstract

**Objective:**

Cathepsin L, a lysosomal endopeptidase expressed in most eukaryotic cells, is a member of the papain-like family of cysteine proteases. Although commonly recognized as a lysosomal protease, cathepsin L is also secreted and involved in the degradation of extracellular matrix proteins. Previous studies demonstrated that the secretion of cathepsin L was stimulated by basic fibroblast growth factor (bFGF) and bFGF-enhanced axonal terminal sprouting of motor neurons. Based on these results, although it has never been directly investigated, we hypothesized that extracellular cathepsin L may induce axonal growth.

**Results:**

To confirm the hypothesis, the axonal growth activity of recombinant cathepsin L was evaluated in cultured cortical and spinal cord neurons. Treatment with recombinant cathepsin L significantly enhanced axonal growth, but not dendritic growth. This result indicated that extracellular cathepsin L may act as a new neuronal network modulator.

## Introduction

Cathepsin L, a lysosomal endopeptidase expressed in most eukaryotic cells, is a member of the papain-like family of cysteine proteases. Cathepsin L plays a major role in antigen processing, tumor invasion and metastasis, bone resorption, and turnover of intracellular and secreted proteins involved in growth regulation. Although commonly recognized as a lysosomal protease, cathepsin L is also secreted and involved in degradation of extracellular matrix proteins (laminins, fibronectin, collagens I and IV, elastin, and other structural proteins of the basement membrane) [1, 2].

Cathepsin L is expressed in almost all tissues, including the brain [3]. In the brain, cathepsin L is expressed in neurons [4], astrocytes [5], and microglia [5]. Among subtypes of cathepsin B, D, E, and L, only cathepsin L showed the drastic age-related reduction in the rat brain [6]. Cathepsin L-deficient mice only demonstrated a reduction in CD4^+^ T cells [7] and recurrent hair loss [8], while cathepsin B-null mice did not have an obvious phenotype [9]. This could be attributed to a compensation for each other due to overlapping substrate specificity. Double knockout mice of cathepsin L and cathepsin B clearly demonstrated a brain-specific phenotype, such as neuronal death in the cerebral cortex and cerebellum, an increase in hypertrophic astrocytes, and axonal degeneration, which resulted in death in infancy [10]. Although this study suggested that the function of cathepsin L and cathepsin B might be necessary for brain development, the specific role of extracellular cathepsin L on neurons remains to be elucidated.

The secretion of cathepsin L is stimulated by various factors. Gene transfer of bFGF or recombinant bFGF treatment in skeletal muscle significantly increased secretion of cathepsin L [11]. Another study demonstrated that a bFGF injection in the gluteus muscle in mice enhanced axonal terminal sprouting of motor neurons [12]. Based on these previous studies, we hypothesized that extracellular cathepsin L may induce axonal growth. To confirm the hypothesis, axonal growth activity of recombinant cathepsin L was evaluated in cultured cortical neurons and spinal cord neurons.

## Main text

### Methods

All animal experiments were performed in accordance with the Guidelines for the Care and Use of Laboratory Animals of the Sugitani Campus of the University of Toyama. The Committee for Animal Care and Use of the Sugitani Campus of the University of Toyama approved all the protocols in the current study. The respective approval number for animal experiments is A2014-INM1. Every effort was made to minimize the number of animals used. All mice were housed in a controlled environment (25 ± 2 °C, 50 ± 5% humidity, 12-h light/dark cycle starting at 7:00 a.m.), with free access to food and water.

### Primary culture

Embryos were removed from ddY mice (Japan SLC, Shizuoka, Japan) at 14 days of gestation. The cortices or spinal cords were dissected; the dura mater was removed using micro tweezers. The tissues were minced, dissociated, and grown in cultures with neurobasal medium (Cat. No. 21103-049, Invitrogen, Grand Island, NY, USA), which included 12% B-27 supplement (Cat. No. 17504044, Invitrogen), 0.6% d-glucose (Cat. No. 049-31165, Wako Pure Chemical Industries, Osaka, Japan), and 2 mM l-glutamine (Cat. No. 078-00525, Wako Pure Chemical Industries), on 8-well chamber slides (Cat. No. 354118, Falcon, Franklin Lakes, NJ, USA) coated with 5 μg/ml poly-d-lysine (Cat. No. 168-19041, Wako Pure Chemical Industries), at 37 °C, in a humidified incubator with 10% CO_2_. The seeding cell density was 2.9 × 10^4^ cells/cm^2^.

### Measurement of axonal and dendritic densities

The density of axons and dendrites were measured at 3 days after culture. Cells were treated with recombinant mouse cathepsin L (Cat. No. 1515-CY, R&D Systems, Minneapolis, MN, USA) for 4 days. The neurons were fixed with 4% paraformaldehyde (Cat. No. 162-16065, Wako Pure Chemical Industries) for 90 min and were immunostained with a monoclonal antibody against phosphorylated neurofilament-H (pNF-H; dilution 1:250, Cat. No. 835601, SMI-35R, Covance, Dedham, MA, USA) as an axonal marker. A polyclonal antibody against microtubule-associated protein 2 (MAP2, dilution 1:2000, Cat. No. ab32454, Abcam, Cambridge, UK) was used as a dendritic marker. The first antibody reaction was performed in PBS containing 5% normal goat serum (Cat. No. 143-06561, Wako Pure Chemical Industries) and 0.3% Triton X-100 (Cat. No. 168-11805, Wako Pure Chemical Industries). Alexa Fluor 488-conjugated goat anti-mouse IgG (dilution 1:300, Cat. No. A-11029, Thermo Fisher Scientific, Waltham, MA, USA) and Alexa Fluor 594-conjugated goat anti-rabbit IgG (dilution 1:300, Cat. No. A-11012, Thermo Fisher Scientific) were used as the secondary antibodies. Nuclear counterstaining was performed using DAPI (1 μg/ml, Cat. No. D9542, Sigma-Aldrich, St. Louis, MO, USA). Fluorescent images were captured with a 10× objective dry lens (Plan-Apochromat, Carl Zeiss, Oberkochen, Germany) using a charge-coupled device camera (AxioCamMRm, binning set at 1 × 1, Carl Zeiss) on an inverted microscope (AxioObserver Z1, Carl Zeiss). A total of 12–20 images were captured per treatment. The lengths of the pNF-H-positive axons and MAP2-positive dendrites and were measured using a MetaMorph analyzer (Molecular Devices, Sunnyvale, CA, USA), which automatically traces and measures the neurite length without measuring the cell bodies. The sum of the axons or dendrite length was divided by the number of MAP2-positive neurons, which were counted by the MetaMorph analyzer.

### Real time-polymerase chain reaction (PCR)

Total RNA was extracted from the cells using TRIsure™ (Cat. No. BIO-38032, Nippon Genetics, Tokyo, Japan), according to the manufacturer’s instructions. The concentrations of the obtained total RNA were measured at 260 nm and the spectrum peak patter was used to evaluate the quality of total RNA. Single-stranded complementary DNA (cDNA) was produced from total RNAs (0.5 μg), using an incubation of 50 pmol of random 6-mer primer and 50 units of reverse transcriptase, in 10 μl of reaction solution, at 42 °C for 15 min, according to the manufacturer’s instructions (ExScript RT reagent kit; Cat. No. RR035A, Takara, Shiga, Japan). A total of 1 µl of the obtained cDNA solution was used as a real-time PCR template. Specific primers for cathepsin L (Accession No. AF121837), i.e., AATACAGAGCCGAGTTCGCT (forward) and TAGAACTGGAGAGACGGATG (backward), were used to produce a 142-bp product. The PCR mixture contained cDNA solution (0.1, 1.0, 10, 100, or 1000 nl) and 0.5 μM of each primer in the reaction solution, with a premix of Taq DNA polymerase and SYBR Green (Cat. No. RR820, Takara). PCR was performed by using a real-time PCR machine (Takara Thermal Cycler Dice; Cat. No. TP600, Takara), with the following: (1) 95 °C for 30 s; (2) 40 cycles of 95 °C for 5 s, 55 °C for 10 s, and 72 °C for 20 s. A linear calibration standard line was obtained using the values of the control treatment. Figure 2 demonstrates the expression values, which were quantified at 100 nl of cDNA solutions; there were three cells in each group.

### Statistical analysis

**Fig. 1:**
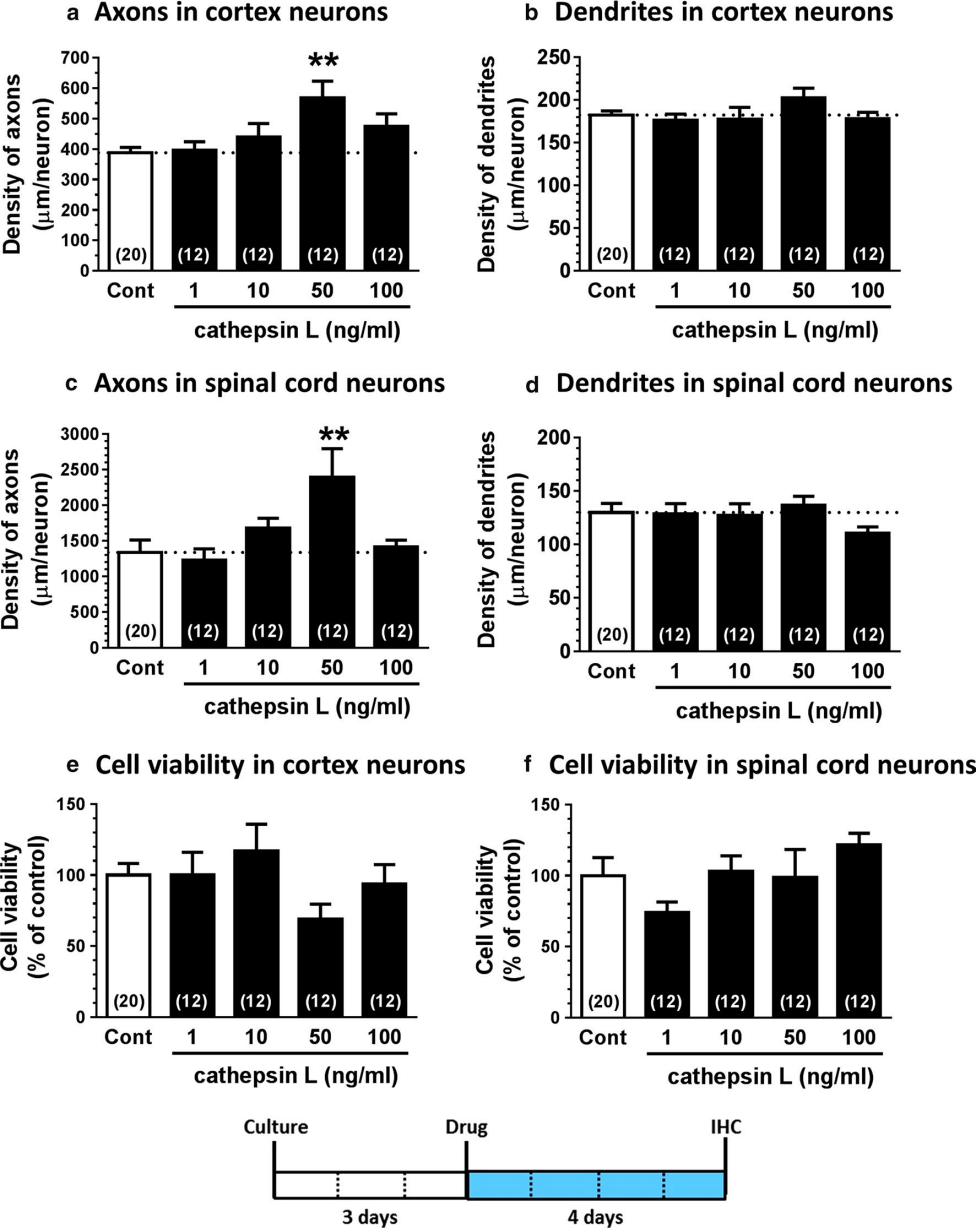
Effect of extracellular cathepsin L on densities of axons and dendrites. Cortical neurons (**a**, **b**, **e**) and spinal cord neurons (**c**, **d**, **f**) were cultured for 3 days and then treated with vehicle solution (Cont) or cathepsin L (at a dose of 1, 10, 50, or 100 ng/ml). The cells were fixed and double-immunostained for against phosphorylated neurofilament-H (pNF-H) and MAP2 antibodies, 4 days after the treatment. The density of pNF-H-positive axons (**a**, **c**) and microtubule-associated protein 2 (MAP2)-positive dendrites (**b**, **d**) were quantified (**p < 0.01, one-way ANOVA with post hoc Dunnett’s test, n = 12–20 photos). The numbers of cortical neurons (**e**) and spinal cord neurons (**f**), which were MAP2-positive, were quantified (one-way ANOVA with post hoc Dunnett’s test, n = 12–20 photos)

**Fig. 2:**
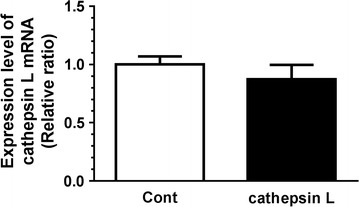
Effect of extracellular cathepsin L on the expression level of cathepsin L mRNA in cortical neurons. Cortical neurons were cultured for 3 days and then treated with vehicle solution (Cont) or cathepsin L (50 ng/ml). Cell lysates were prepared for cathepsin L mRNA quantification 4 days after treatment. The expression levels are shown as a relative ratio to the control group. n = 3 wells

Statistical comparisons were performed using a one-way analysis of variance (ANOVA) with a post hoc Dunnett’s test (Fig. 1), and unpaired two-tailed *t*-tests (Fig. 2), using GraphPad Prism 6 (GraphPad Software, La Jolla, CA, USA). Statistical significance was set at p < 0.05. Data are presented as mean ± standard error (SE).

### Results

We evaluated the effect of recombinant cathepsin L on axonal growth and dendritic growth of cultured cerebral cortex neurons and spinal cord neurons. The pro form of the recombinant mouse cathepsin L, which consisted of a signal peptide (residues 1–17), pro-region (residues 18–113), and mature chain (residues 114–334), was used in the current study since secreted cathepsin L exists mainly in the pro form [5]. This form is inactive [13]. Autolysis at pH 3.0 produces the mature form of this enzyme, activating own. However, there was no activation at pH 6.5 or higher [13]. This meant that the physiological conditions of the culture experiments in the current study was unable to trigger the enzymatic activation of the recombinant cathepsin L. Therefore, we aimed to evaluate the effect of extracellular cathepsin L on axonal growth activity, independently of its enzymatic activity. Cathepsin L was added to neurons, after 3 days in culture, at doses of 1, 10, 50, and 100 ng/ml. After 4 days of treatment, axons and dendrites were immunostained using antibodies against specific marker proteins. Compared to vehicle solution treatment (Cont), the administration of cathepsin L (at 50 ng/ml) significantly increased the density of axons in cortical neurons (Fig. 1a, 95% confidence interval of difference; − 301.2 to − 59.92) and spinal cord neurons (Fig. 1c, 95% confidence interval of difference; − 1783 to − 321.9). In contrast, cathepsin L treatment did not alter the density of cortical (Fig. 1b) and spinal cord (Fig. 1d) neurons. The viability of neurons was evaluated by counting the MAP2-positive neurons. Cathepsin L did not significantly change the numbers cortical neurons (Fig. 1e) and spinal cord neurons (Fig. 1f), at any dose. These data indicated that cathepsin L plays a role in axonal growth, without any toxicity.

Cathepsin L could be distributed and work in various organelles, such as the lysosome, cytosol, nucleus, and extracellular matrix. Although the applied recombinant cathepsin L would not have penetrated the neurons, it is possible that extracellular cathepsin L might affect the intracellular expression of cathepsin L. Therefore, the mRNA level of cathepsin L in cortical neurons was measured after a 4-day recombinant cathepsin L treatment. The cathepsin L treatment did not affect the expression level of neurons.

### Discussion

The current study indicated for the first time that extracellular cathepsin L stimulates axonal growth, but not dendritic growth, in cortical neurons and spinal cord neurons (Fig. 1). Since there was no previous data on recombinant cathepsin L treatment of cells, we optimized the appropriate effective doses of the recombinant cathepsin L ourselves, in reference to the concentration of cathepsin L in human serum (i.e., approximately 4 ng/ml) [14]. Therefore, we set the dose range at the ng/ml order. The molecular mechanism of extracellular cathepsin L for axon-specific growth remains unknown. Generally, extracellular cathepsin L is thought to degrade several components of the extracellular matrix, such as laminins, fibronectin, collagens I and IV, elastin, and other structural proteins of the basement membrane [1, 2]. Since there is no previous report demonstrating that degraded extracellular matrix contributes to axonal growth, extracellular cathepsin L may directly stimulate some molecules on neurons to extend axons. In addition, the cathepsin L used in the current study was in the enzymatically inactive pro form, Therefore, the axonal growth that occurred due to cathepsin L might have been independent of conventional endopeptidase activity. Whether there is a novel signal pathway of extracellular cathepsin L, which elicits axonal growth, remains to be clarified in future. Cathepsin L is secreted from various cells, including, for example, skeletal muscles [11]. In the current experiment, cathepsin L treatment did not change the expression level of intracellular cathepsin L mRNA (Fig. 2). Basic fibroblast growth factor (bFGF) treatment or bFGF transfection to skeletal myocytes increased the mRNA level of cathepsin L and its release [11]. In contrast, extracellular cathepsin L itself did not affect the upregulation of cathepsin L mRNA in the current study. Therefore, extracellular cathepsin L may not have increased the secretion of cathepsin L.

A recent report demonstrated that immunoreactivity for cathepsin L was detected in nerve fibers bundles and some nerve cell bodies [15]. In the cerebral cortex and subcortical structures, the expression of cathepsin L was very high in white matter regions, such as the corpus callosum. These results may suggest that cathepsin L in neurons is related to axonal formation. Therefore, we postulated that cathepsin L may have a novel role as an axonal growth facilitator, which is involved in neuronal circuit formation.

## Limitations

- Extracellular cathepsin L stimulate axonal growth in cortical neurons and spinal cord neurons.
- The mechanism by which cathepsin L-induced axonal growth remains unknown.
- In vivo evidence demonstrating cathepsin L-mediated axonal growth has not been obtained yet.

## Abbreviations

bFGF: basic fibroblast growth factor
cDNA: complementary deoxyribose nucleic acid
MAP2: microtubule-associated protein 2
PCR: polymerase chain reaction
pNF-H: phosphorylated neurofilament-H
